# Actual usage assessment among cloud storage consumers in the Philippines using a machine learning ensemble approach

**DOI:** 10.1038/s41598-024-80676-9

**Published:** 2024-11-22

**Authors:** Ardvin Kester S. Ong, Gerlyn C. Altes, Josephine D. German

**Affiliations:** School of Industrial Engineering and Engineering Management, Mapúa University, 658 Muralla St., Intramuros, Manila, 1002 Manila, Philippines

**Keywords:** Cloud storage, Actual use, Machine learning, Technology acceptance model, Valence framework, Applied mathematics, Computational science, Computer science, Information technology

## Abstract

Cloud storage has been widely considered among developed and developing countries due to its ability to provide a platform for large data and information storage. Developing countries like the Philippines have started using this storage and have only since considered the free services. With the aim to understand utility for development and continuous patronage, there has been lacking evidence in the intention and actual use of cloud storages. The need for study is evident to promote and develop concrete strategies for cloud storage uptake, even if payment is needed for extra storage. This study analyzed the antecedents of actual use behavior of cloud storage in a developing country like the Philippines using a machine learning ensemble (MLE). With 616 valid responses, a total of 33,264 datasets were processed to analyze the actual use of cloud storage among Filipinos, measured using the integrated extended technology acceptance model and valence framework. With an average accuracy of 93% and 90% for the MLE considered, results have presented consistent output of voluntariness, subjective norm, perceived benefit, perceived usefulness, and perceived ubiquity to be contributing factors affecting actual use behavior. It could be posited that both personal and professional usage of cloud storage has been considered by users. In addition, due to people’s readiness to use technology nowadays, the adoption of which is relatively convenient for them. Evident from the findings, further technological infrastructure is needed to be enhanced in the country for a more positive continuous intention. Therefore, the application of the integrated framework may be used and expanded for other technology utilities in different countries. Lastly, practical and managerial insights were built on the results to provide strategies and development needed for marketing, utility, and application.

**Supplementary Information:**

The online version contains supplementary material available at 10.1038/s41598-024-80676-9.

## Introduction

Current resources are highly reliant on data. Different sectors have established the need to store information, data, and file – may it be for personal or business endeavors. To which, physical storage has been evidently insufficient and inefficient in terms of storage in general and cost. Aside from storage, Yang et al.^1^ established the utility of online storage (cloud storage) as a technological trend which is used for transformation of data to information. Sharma et al.^2^ explained that the dominance of data usage nowadays presented the need for cloud storage due to the need for innovation, digital transformation, and even processing. The increase of cloud storage has therefore increased the different variety, ranging from Google drive, Apple iCloud, Dropbox, Amazon Cloud Drive, and many more.

Singh^3^explained how the increase of users with cloud provided convenience and efficiency among users. To which, more and more people in different countries have utilized the storage for its usability and accessibility, automation, file-sharing, cost, security, scalability, automation, synchronization, user accommodation, and even disaster recovery. Vailshery^4^showed a steady increase of users for the technology, with 1.1 million users in 2014 to 2.3 million in 2022. The increase of users also prompted developers to invest and profit from the technology^5^. A lot of development has been seen with the technology since its development to cater to different purposes of cloud storage such as cloud computing^6^. However, the analysis of user’s behavioral intention has been neglected by researchers – most of which only focused on analysis of the usage and application of the technology.

The need to assess the user’s behavioral intention and actual use is needed in order for continuance in the development of the technology. As technology continuously develops, is being enhanced, and is relatively used, there is a clear research gap in an overall perspective as the technology is highly utilized and considered in the current generation. Yet, since its development, the acceptance and use of technology has remained limited. On the other hand, Kim et al.^7^explained that the advancement of technology would build undeniable competition, and marketing strategies and promotion would be difficult to develop if people have become accustomed with one type. Usual neglect of consumer analysis probed to be a problem in different industries, especially in the technology sector^8^. In light of the continuous development, the need for consumers to engage and promote the uptake of the technology would be needed for any development and enhancement. Chesbourgh et al.^9^ expounded on the commercialization of technology for consumer utilization. Their study presented that different countries and its users would enable different ideas, promotions, manufacturing, and product selling. Thus, the need to further analyze and classify factors affecting behavioral intentions and actual use of technology should be assessed, mainly to help developers and manufacturers sell the product.

In developing countries, technological advancement is more so in need of analysis. This is because their communities are building on societal and country development. Before the start of product development, consumers in developing countries should be considered since their behavioral aspect when it comes to technology usage are not quite accustomed compared to developed countries^9,10^. Given this circumstance, the need for establishing utility, uptake through intention and actual use, and technology acceptance is needed to be deciphered simultaneously. Despite the challenges brought by technology usage, developing countries have tried to be at par with the advancement of technology since recent events such as the COVID-19 pandemic pushed the different sectors to use online resources^11^. Therefore, developing countries such as the Philippines also adopted technology usage, such as that of cloud storage for both personal, education, and business use.

Recent studies have not focused on the behavioral aspects of people when it comes to cloud storage and cloud technology. Moreover, most of which focused on usage, advancement, and development. Ren et al.^12^ explored the use of cloud storage in households, specifically smart homes. Their analysis focused on the development of technology due to lack of security features, relational database security which is fundamental in households. Fan et al.^6^ explained the development of cloud computing, focusing on the data management, convenience, its maintenance, and cost effectiveness. The study proposed a security system for effective data protection available for users. Xue et al.^13^ dealt with the collaborative access of cloud storage and assessed the current access permission by cloud storage. Yan et al.^14^focused on the remote access of cloud storage and criticized current security system development. Krumm and Hoffman^15^ explored the costs for clinical usage of cloud storage, showing a total cost reduction with rapid file transfer. Widjaja et al.^16^ focused on the willingness of users to utilize cloud storage, focusing on developed countries in Asia. The results of their study showed that security risks through privacy issues were greatly considered in relation to perception of cost effectiveness. Culture also played a role in the privacy context of users, which should be explored more. Lastly, Syed et al.^17^ reviewed recent practices, security risks, and measures for cloud storage. There are other studies that have been published, but more or less dealt with technology advancement and current problems. To much of the author’s identification, no studies have covered the user’s behavior and perspective on actual use of cloud storage, specifically in developing countries such as the Philippines.

The goal of this study was to assess the actual use of cloud storage among users in the Philippines through machine learning ensemble (MLE). To which, the integration of different established theories was considered such as the extended technology acceptance model (e-TAM) and the valence framework to provide a more holistic analysis of behavioral intentions and actual use. The need for analysis using MLE was accounted for due to limitations and disadvantages presented by traditional statistical analyses and multivariate tools. Fan et al.^18^expounded on the limitations of powerful multivariate analysis such as structural equation modeling (SEM). Their study prompted a result indicating that some analysis of SEM may provide little to no significance when mediating effects are present due to the interrelationship of latent variables. This was a justification, similar to the study of Woody^19^. MLE’s therefore have been widely utilized to assess human behavior, either solely^11,20^or integrated with multivariate tools^10,21^ for better analysis with justification.

The results of this study would provide insights and benchmark on user’s actual use of cloud storage. This will help developers and technology industries understand their user’s behavioral aspects – which could be a basis on the interface and system development. From a theoretical standpoint, the framework developed in this study may be considered for analysis of technologies and systems currently present for user actual usage. The results of this study could therefore be a basis for the development and promotion of use of cloud storage. Moreover, enhancement and modifications could be made based on the findings of this study, which may be considered by different cloud storage industries worldwide.

## Related literature and conceptual Framework

### Cloud Storage and Cloud Computing

Similarities among cloud computing and cloud storage made the two synonymous. However, the distinction of which is evident. As explained by several studies^22–24^, cloud computing is based on layers which considers pay-per-use. The different layers of software, platform, and infrastructure as service provides the users software which are cloud-based from a certain developer which sells the product. Among the softwares may be Google Apps, LinkedIn, Microsoft Office, and the like; under the platform may be Google, Amazon Web Services, LongJump and the like, which offers services for users to manage, develop, and execute applications. Lastly, under infrastructure may be the Amazon Web Services EC2 and S3 which provides users with resources of hardware, servers, storage, and even networking. Overall, cloud computing enables ubiquitous usage, network access on demand, convenience, and shared pool data and information^25^.

The difference for cloud storage is that it is an architecture which transmits data either privately, remotely, or shared^22^. It also provides back up, maintenance, and availability for flexible coordination and transaction among users. Compared to cloud computing, cloud storage is a pay per month usage which can be used by specific organizations or companies. The common online storage people usually utilize are under the cloud storage, wherein free data is given for consumers by the different platforms. To which, they are deciphered as public, private, community, and hybrid cloud storage dependent on its intended usage^23–26^. Despite the differences, its main purpose still remains the same, storage, development, sharing, and automation among users intent.

### Theories for technology usage and hypotheses

Different studies have argued which framework should be considered for the assessment of technology among its users. Venkatesh et al.^27^ have developed the unified theory of acceptance and use of technology (UTAUT2) – which is a framework that is established to assess actual use and acceptance of technology. However, different studies such as that of Yuduang et al.^28^ justified that UTAUT2 is used to assess only new technologies, systems, and usage in new environments. Other theories have been said to be better for analysis of technologies and systems when users are aware and have been accustomed to using it. One of which is the technology acceptance model (TAM).

TAM is a framework that has been utilized widely by different studies in the assessment of technology and consumer’s actual use. Venkatesh and Bala^29^have established the use of TAM, developing and extending the theory to different applications. The most basic and common TAM model considers the perceived ease of use, perceived usefulness, behavioral intentions, and actual use^30^. Other studies have argued that attitude may or may not be considered in the framework for assessment when dealing with technology acceptance^31^. To which, other studies have also justified the extension of TAM with exogenous latent variables^32^. This study has therefore considered the extension of TAM with other latent variables and integration with the valence framework.

As explained in the model developed by Venkatesh et al.^27^, technology usage is usually influenced by surrounding people. It was also explained by Schepers and Wetzels^33^. That the tendency of humans to interpret the perception of technology usage is affected by confirmation from others. To which, it affects the overall behavioral intention and actual use of a system. On the other hand, Yuduang et al.^10,28^explained how the relevance of technology usage to everyday lives affects people’s perception of its use. However, this effect may go positively or negatively if the relevance of technology is not applicable. In addition, Tripathi^34^ expounded on the relevance of jobs needed to be accomplished with the help of technology. The ease of job accomplishment reflects a positive perception on the usage, therefore having a positive and significant effect on behavioral intentions and actual use. In the case of this study, workmates, classmates, colleagues, or friends were hypothesized to affect the behavioral intentions and actual use of cloud storage; and job relevance would promote a positive outcome as:

**H1.** Subjective norm has a positive effect on TAM.

**H2.** Job relevance has a positive effect on TAM.

The proposed extension of TAM by Obeidat and Turgay^35^showed that both job experience and voluntariness to use technology are significant latent variables affecting behavioral intention and actual use. Their study showed a positive perception on the technology usage, both on ease and its utility – which is why people accepted it. When people are inexperienced with the technology at hand, negative outcomes are evident, leading to no actual use^36^. For which, a positive outlook would lead to voluntariness and habit for continuous usage of a technology^28^. Proactive actions among users would promote the continuous usage of a technology^37,38^. The study of Mohiuddin et al.^39^ explained that the voluntariness would be a deficit if mandated with the use of technology. Since open access for cloud storage and its utility is available, the following were hypothesized:

**H3.** Experience has a positive effect on TAM.

**H4.** Voluntariness has a positive effect on TAM.

With the indicated usage of TAM, the different hypotheses were built which are evident in the basic technology acceptance model^27,29,31,40^:

**H5.** Perceived usefulness has a positive effect on behavioral intentions.

**H6.** Perceived ease of use has a positive effect on behavioral intentions.

**H7.** Behavioral intentions have a positive effect on actual use.

The valence framework (VF) has been discussed to provide aspects of consumer decision, intention, and acceptance which was developed in 1975 by Peter and Tarpey. Several aspects are considered such as perception of overall benefits and risks, ubiquity, and cost^34^. The study of Song et al.^41^justified the use of valence framework when technology is considered. They explained that drawbacks and advantages of technology usage are evaluated with the use of the valence theory as both positive and negative aspects are variables considered which can then be evaluated for consumer decision behaviors. To which, technology studies relating to health^42^, online payment through mobile phones^43^, e-commerce^44^, and protocols in internet usage^45^ have been assessed using the VF. Therefore, the advantages and disadvantages of the technology, cloud storage, together with the consumer’s actual use was evaluated in this study using the integrated eTAM and VF. Thus, it was hypothesized that:

**H8.** VF latent variables have a positive effect on TAM.

### Conceptual framework

**Fig. 1:**
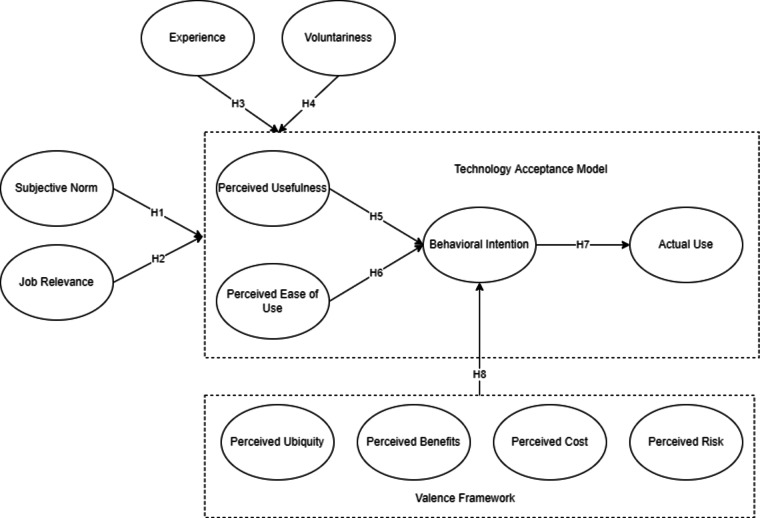
Conceptual Framework.

The conceptual framework through the integration of eTAM and VF is presented in Fig. 1. It could be seen that TAM was extended with different latent variables such as experience, voluntariness, subjective norm, and job relevance^29,40^. Evident from the study of Yaseen et al.^46^, the organizational aspect should be considered (in this study, job relevance) since this area implicated a positive impact on behavioral intention to use cloud computing adoption. VF framework considered four latent variables of positive and negative applying the suggestion seen from the study of Tripathi^34^. A total of 8 hypotheses were created wherein 4 came from the extension of TAM, 3 under TAM, and 1 from the effect of VF to the TAM.

### Methodology used among related studies

Based on the collated literature, several methodologies and assessment tools have been considered for analysis. Most commonly, multivariate analyses such as structural equation modeling and machine learning algorithms have been widely utilized. Summarized in Table 1 are the comparisons among related studies.

**Table 1:** Comparison from related studies.

Author	Tool	Framework	Output	Limitation
Venkatesh et al. ^27^	Structural Equation Modeling	UTAUT	Further variables are needed to be considered, extended, or added for a more holistic assessment.	Several constructs may be deemed insignificant due to cross-loading effects.
Yuduang et al. ^28^	Structural equation modeling and neural network	UTAUT2	Consistent output on both tools was proven. The algorithm was suggested to be improved.	Other machine learning algorithms could be considered and utilized.
Venkatesh and Bala^29^	Structural Equation Modeling	TAM 3	Further variables are needed to be considered, extended, or added for a more holistic assessment.	Several constructs may be deemed insignificant due to cross-loading effects.
Pal and Vanijja^30^	Descriptive and Structural Equation Modeling	System Usability Scale and TAM		
Prasetyo et al. ^31^	Structural Equation Modeling	TAM and Delone and McLean IS Success Model		
Zheng and Li ^32^	Structural Equation Modeling	Extended TAM	Positive and significant output was obtained from the analysis on all the relationships.	Additional variables are suggested to be included as one theory could not further measure the overall intention.
Schepers and Wetzels^33^	Correlation and Structural Equation Modeling	Extended TAM	Additional tests were needed to encompass the path analysis.	Correlation analysis needed support for holistic assessment.
Tripathi^34^	Exploratory Factor Analysis and Structural Equation Modeling	Valence Framework and TAM	Additional tests were needed to encompass the path analysis and further variables were needed to be considered, extended, or added for a more holistic assessment.	Limited insights and implications were seen based only on the relationship of the path analysis.
Obeidat and Turgay^35^	Correlation	Social Exchange Theory and TAM	Positive implications were made based on the correlation.	Relationship was limited to each construct rather than an overall evaluation.
Purnama and Ginardi^36^	Descriptive statistics, correlation, and regression	TAM 2	Further variables are needed to be considered, extended, or added for a more holistic assessment.	Several constructs may be deemed insignificant due to cross-loading effects and that relationship was limited to each construct rather than an overall evaluation.
Yaseen et al.^46^	Multiple linear regression	Technology Organization Environment and Diffusion of Innovation		Limited with the constructs used, model verification was needed to be explored, and a mixed method was suggested to be employed.

Evident from related studies, limitations on the tool, framework, and overall assessment were seen. That is, a pattern on framework extension and integration, limitations on the construct based on the path analysis, and relationship assessment were evident. As suggested by related studies mentioned, further and more powerful tools could help in the development and assessment of significant factors under human factors and consumer behavior^11,27,28^.

## Methodology

### Participants

For the data collection process, German et al.^11^presented that 400 respondents would represent the generalized findings. This was based on the Yamane Taro calculation of 62.6 million Filipinos in the country. With 95% confidence interval, 400 sample size is said to encompass a generalized insight among respondents (Eq. 1). As support, a survey conducted by Statista in 2022 presented that only 11% of Filipinos utilizes cloud storage^47^. To reach a potential increase among users, only a 5% error rate on the total respondent’s generalizability was considered to obtain higher response rate.

1 $$\:n=\frac{N}{1+N\left({e}^{2}\right)}$$

*Wherein: N = 62*,*600*,*000 Filipinos*.

*e = 0.05 to account for 5% error*.

*n resulted to 399.99.*

This study was able to collect 616 valid respondents. Through convenience sampling, the distribution of descriptive statistics is represented in Table 2. A filtering question of whether they use cloud storage depicted valid respondents. The data collection period ran from November 2022 until February of 2023. To which, the majority were male (56.66%) and the rest were female (43.34%) with ages from 15 to 28 (33.28%) and 29–38 years old (46.43%) as majority of the respondents. Almost half of the respondents are in college (43.34%), closely in high school (21.92%) and working class (27.11%), while the remaining are retired already (7.630%). As explained by Widjaja et al.^16^, most users of cloud storage are typically male who are within 29–38 years old in both education and working class. Similar to the collected data from this study, it could be deduced that information and data needed for each sector could easily be shared and stored in cloud storages. Perceived benefit, trust, and perceived cost played a role as to why people would have an intention.

However, the privacy issues were raised by Widjaja et al.^16^. With most of their study representing the government workforce and private companies, privacy issues were highly relevant with regards to the use of cloud storage for sensitive data; issues similar with Syed et al.^17^. The difference for this study was that this was conducted in a developing country, the Philippines. Perception to use of technology was discussed to be different in different types of countries^9^. Thus, further analysis for difference is needed. In accordance, most of the respondents have moderate (55.52%) to strong (24.19%) internet connection. The internet connection projects a difference in perception among users, which also affects their actual use^31^. Moreover, the respondents are within the lower bracket of monthly salary or allowance; with less than 15,000 Php (35.39%) as the highest, followed by 30,001–45,000 PhP (26.62%), and 15,001–30,000 PhP (24.84%). Lastly, most of which are located in urban areas (76.79%) compared to rural areas (23.21%).

**Table 2:** Demographic Profile (*n* = 616).

Demographics	Characteristics	Distribution	Percent
Gender	Male	349	56.66
	Female	267	43.34
Age	15–28 years old	205	33.28
	29–38 years old	286	46.43
	39–48 years old	86	13.96
	49–58 years old	30	4.870
	59 years old and above	9	1.460
Education/Occupation	High School	135	21.92
	College	267	43.34
	Working Class	167	27.11
	Retired	47	7.630
Access to Internet	Weak	125	20.29
	Moderate	342	55.52
	Strong	149	24.19
Monthly Income/Allowance	Less than 15,000 PhP	218	35.39
	15,001–30,000 PhP	153	24.84
	30,001–45,000 PhP	164	26.62
	45,001–60,000 PhP	43	6.980
	60,001–75,000 PhP	26	4.220
	Higher than 75,000 PhP	12	1.950
Location	Urban	473	76.79
	Rural	143	23.21

### Questionnaire

The questionnaire utilized in this study was adapted from different studies, covering related latent variables and theories. To encompass the unmeasured variables that were considered in this study, a total of 54 adapted items were considered as seen in the supplementary files. From which, the 12 latent variables were measured and prompted a total of 33,264 (54 × 616) data points considered in this study.

The measure items, data collection process, and analyses were approved by the institutional review and ethics committee boards of Mapua University (FM-RC-23-01-31). The informed consents were obtained from participants, and they have the right to withdraw any time they felt uncomfortable. The informed consents were provided before the initiation of the fieldwork, recorded at FM-RC-23-02-31. The statements of anonymity and secrecy had been completed before the beginning of the survey. All study procedures were conducted in accordance with the principles of the Declaration of Helsinki.

### Machine learning algorithm

Machine learning algorithms are typically utilized for analysis of large datasets due to its capability to generate a high accuracy classification model^28^. In addition, it was explained that a lot of behavioral studies currently have been utilizing the ensemble of machine learning due to the limitations of SEM^11,20^. Its ability to analyze nonlinear relationship constructs have promoted its utilization among related studies. Chen et al.^48^ utilized MLE to analyze risk evaluation for flood disaster, focusing on behavior of people in China. Their results justified that the use of random forest classifier has been exemplar compared to the basic decision tree when it comes to classifying datasets. The study of Andres et al.^49^ showed that neural networks are more sophisticated to create a classification model for data analysis, focusing on developing and emerging economies. The pattern available in the different datasets considered can easily be analyzed with neural networks such as deep learning. To which, an ensemble of both random forest and deep learning were considered in this study.

Utilizing the Jupyter Notebook v6.4.8, data preprocessing was conducted. Utilizing correlation analysis for the feature selection process, Yuduang et al.^28^ explained that 0.20 correlation coefficient still presents significance which should be the threshold for the items. In addition, the 0.05 p-value was considered for the significance level. Fortunately, all items were deemed significant as to why data aggregation was employed to represent the 11 latent variables that served as the inputs for the MLE. Min_max scalar was considered as a package of the Jupyter Notebook for the data normalization process prior to the initial optimization process. The parameters of random forest were considered, similar to the study of German et al.^11^ when it comes to the criterion and splitters, test ratios, and tree depth. Similarly, the deep learning neural network parameters and optimization process was adopted from the study of Ong et al.^20^. Each iteration was collected and recorded, performing analysis of variance to determine the significant differences among the averaged results.

## Results

### Random forest classifier

**Fig. 2:**
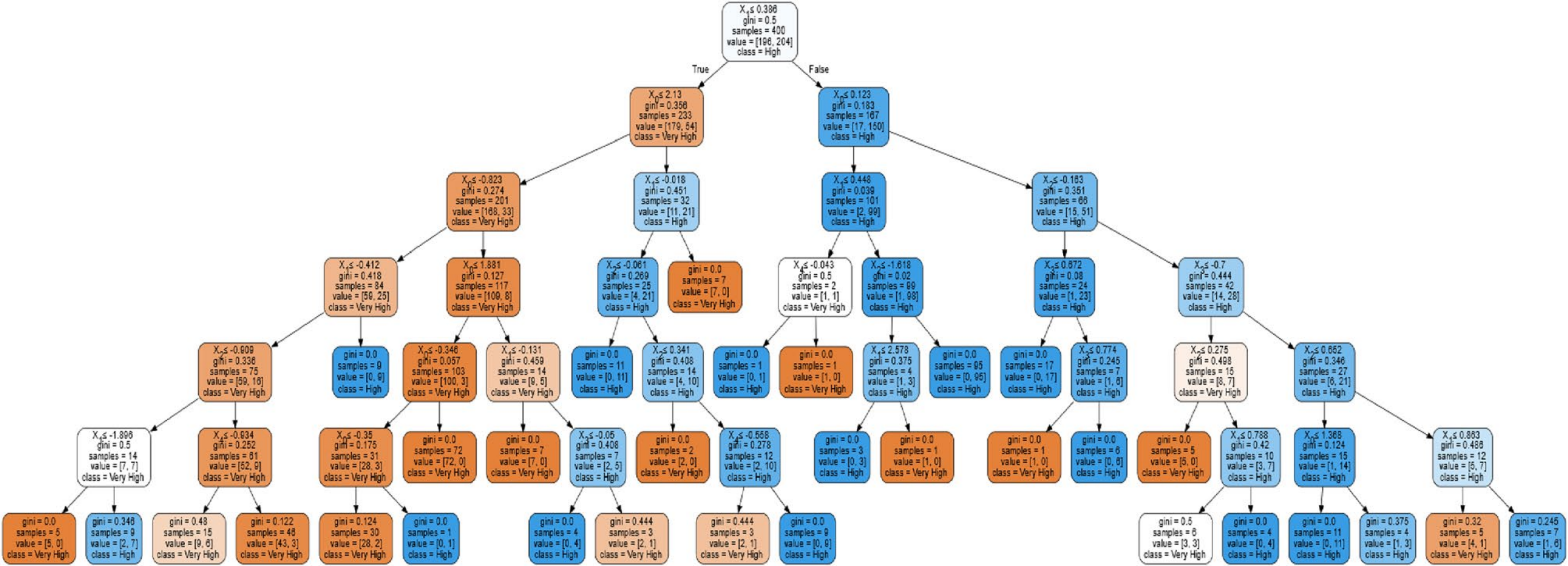
Optimum Random Forest Classifier Output. **X**_**0**_**–** Subjective norm, **X**_**1**_ – Voluntariness, **X**_**2**_ – Perceived ubiquity, **X**_**3**_ – Perceived benefit, **X**_**4**_ – Perceived usefulness.

From the optimization in different tree depths used, 6 presented the most consistent and highest average accuracy of 93%. Presented in Table 3 are the summarized results of the different parameters at depth 6. It could be seen that consistent outputs are seen at the 80:20 training and testing ratio, highest at gini and best criterion and splitter. The following is Fig. 2 which represents the optimum decision tree with random forest classifier.

**Table 3:** Random Forest Classifier Summarized results.

Category	60:40	70:30	80:20	90:10
Random				
Gini	89.45	83.54	79.00	80.04
Standard Deviation	5.523	6.405	0.000	4.679
Entropy	80.41	82.64	84.00	83.12
Standard Deviation	3.636	5.578	0.000	5.578
Best				
Gini	89.00	92.00	**93.00**	91.00
Standard Deviation	0.000	0.925	**0.000**	0.000
Entropy	85.05	81.09	82.00	92.00
Standard Deviation	0.725	0.105	0.000	0.000

As seen from Fig. 2, voluntariness (X_1_) prompted the most influencing latent variable affecting actual use. This would lead to subjective norm (X_0_) with either less than or equal to 2.13 having lower value of X_1_ or 0.123 if X_1_ has greater value. Considering the first implication, X_0_, X_1_, and perceived ubiquity (X_2_) would be considered, leading to very high actual use. On the other hand, if the first child is not satisfied, X_1_ and X_2_ will be considered which will lead to only a high actual use behavior. From which, having high X_2_ will lead to very high actual use. This indicates that the more available the technology for utility is, the more actual use behavior will be positive among users.

On the other hand, if the other child node (X_0_) is satisfied, X_1_ and perceived usefulness (X_4_) will be considered with value less than or equal to −0.043 which will lead to a high actual use behavior. Otherwise, a very high actual use will be evident. It could be posited that less usefulness of technology will not benefit the users. In accordance, if X_0_ will not be satisfied (greater than 0.123), then X_2_ will be considered, and then perceived benefit (X_3_), with value less than or equal to 0.672 – leading to high actual use. Otherwise, X_2_, X_1_ or X_0_ will be considered which will lead to very high actual use behavior among users. In relation to this study, users would want the use of technology to be beneficial and useful in their daily activities.

From the random forest optimum output, it could be deduced that voluntariness and subjective norm dictates actual use behavior among users. This has a very high significance and influence on actual use for cloud storage. In addition, the perceived ubiquity, perceived usefulness, and perceived benefit have a high effect on actual use of cloud storage. To further validate the findings, it was suggested by different studies that neural networks may be applied for further analysis^11,28,50^.

### Deep learning neural network

**Fig. 3:**
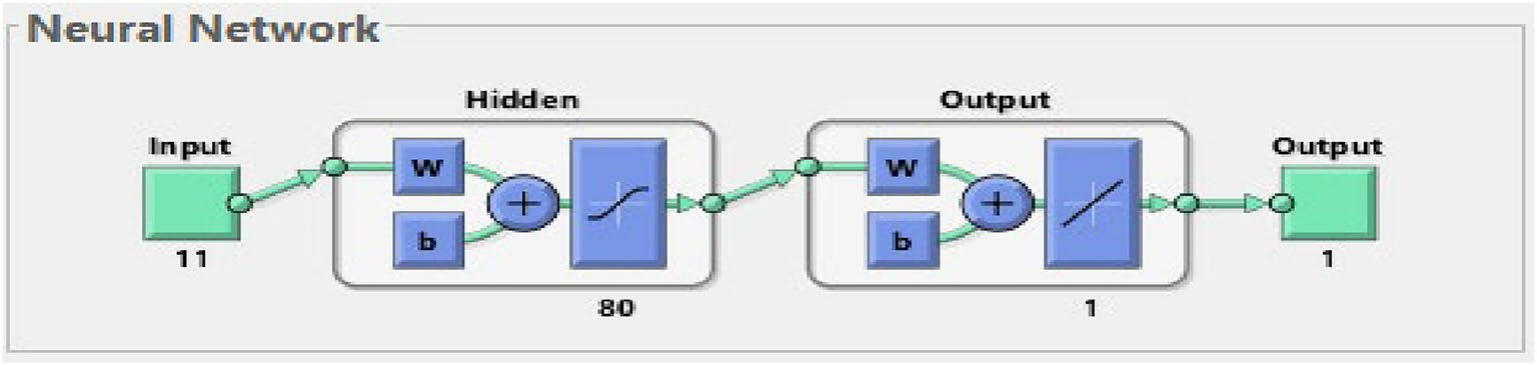
Deep Learning Neural Network Optimum Model.

For the summarized result of the neural network (Figs. 3), 80 nodes presented a high result of 90%. The Levenberg-Marquardt algorithm was considered and utilized with 100 iterations. This type of algorithm was indicated to be effective as presented in the study of Öztürk and Başar^51^. In accordance, the prediction output to identify the error rates and best combination is attached in the supplementary file. Based on the predictive output, the best combination is with 90% training set and 5% for both testing and validation sets. As suggested, this would be utilized for identifying the importance of the different inputs (variables used in this study)^51^.

Depicted in Table 4are the output based on the different training: testing: validation set, where an increase in the training set reduces the error. It could also be seen that the root mean square error (RMSE) of the overall output per set decreases as the training set increases. That is, the 70:15:15 has a predictive error of 9.56% and RMSE output of 0.9121, 7.24% for 80:10:10 with 0.5165 RMSE, and 3.80% error for 90:5:5 and 0.2714 RMSE. Despite the differences in average error output and RMSE, all of which are relatively close and are small. This means that accurate predictions may be obtained with the different sets, sub-optimum on 90:5:5. Presented after is the script used to run the neural network, adopted from the study of Öztürk and Başar^51^.
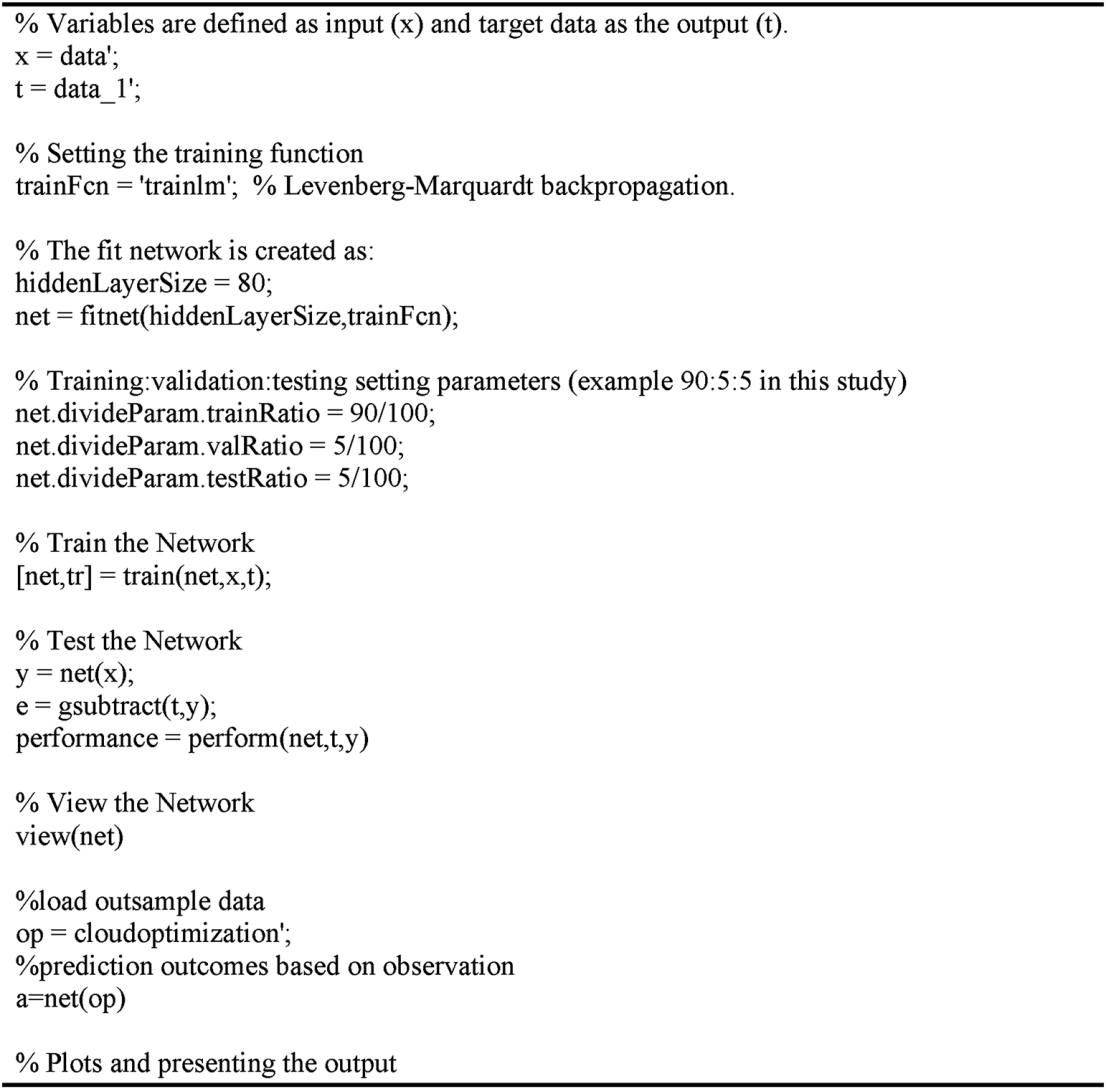


**Table 4:** Prediction output (training: testing: validation).

70:15:15	Error	80:10:10	Error	90:5:5	Error
3.0004	0.01	3.0001	0.00333	3.0004	0.01
2.0002	0.05996	2.0014	0.07	1.9919	0.47467
3.9997	0.0025	3.9998	0.005	4.0068	0.17501
4.9939	31.294	3.8036	23.928	5.1438	35.235
6.9906	2.78178	6.8014	2.021	6.8567	0.81307
5.9918	4.88744	5.7126	4.79	6.0587	6.05854
4.9935	95.4327	2.5551	48.898	5.1038	99.7495
5.4979	9.9668	4.9996	0.008	4.729	5.41243
4.3326	59.4979	2.7164	37.3138	4.3575	60.4145
5.9906	0.05506	5.9939	0.10167	6.6597	11.108
1.9981	0.13495	2.0008	0.04	2.0349	1.70432
7.0002	0.14592	6.99	0.14286	7.0871	1.38913
4.9902	0.15007	4.9977	0.046	5.134	2.72725
5.6689	0.07061	5.6649	0.03118	5.5978	1.18449
4.6669	0.02356	4.668	0.02857	4.6485	0.41774
6.995	0.06151	6.9907	0.13286	7.0474	0.81108
0.9988	0.86352	1.0075	0.75	0.9886	1.87593
7.0724	24.7953	5.6672	0.00941	5.7851	2.08039
7.2147	66.6559	4.3291	0.09769	4.5375	4.81393
6.1193	3.32554	6.3298	0.05579	6.373	0.68249
4.7977	19.993	5.9966	0.05667	6.1766	3.0017
10.2997	81.7936	5.6656	0.01882	5.674	0.14826
5.9935	0.0434	5.9909	0.15167	5.881	1.83445
6.6692	0.22843	6.654	0.19	6.7812	1.91163
4.9958	0.078	4.9997	0.006	5.0857	1.7201
1.3342	0.03749	1.3337	0.0275	1.3483	1.0947
5.0458	25.8964	4.0079	0.1975	4.124	2.89678
6.0004	99.9067	3.0016	0.05333	3.1271	4.1811
5.9946	3.2537	5.8057	3.23833	6.226	7.23944
5.9995	0.16474	6.0094	0.15667	6.1694	2.6625
6.7677	103.015	3.3336	0.008	3.3527	0.57295
5.4929	51.3529	3.6292	34.0145	5.6766	56.4146
5.5692	20.4002	6.9965	0.05	6.9329	0.90903
5.9985	0.10681	5.9921	0.13167	6.2189	3.78498
5.0997	27.0951	6.995	0.07143	7.1779	2.61472
5.0212	16.2547	5.9958	0.07	6.041	0.75386
6.9885	0.06013	6.9843	0.22429	7.0511	0.95643
6.3261	0.00474	6.3258	0.11895	6.5187	3.04942
6.9871	0.29112	7.0075	0.10714	7.0794	1.02604
5.0002	0.1001	4.9952	0.096	5.0876	1.84978
5.3297	10.4117	5.9491	11.5456	5.4163	8.95598
5.9484	48.6914	4.0005	0.0125	4.1933	4.8194
4.9976	31.1018	3.812	23.76	5.0858	33.4155
2.3314	52.9628	4.9565	112.421	2.391	51.7603
3.9959	0.12248	4.0008	0.02	4.1479	3.67676
3.9965	0.18981	4.0041	0.1025	4.1338	3.23918
2	0.04998	2.001	0.05	2.0043	0.16492
5.6626	0.03178	5.6644	0.04	5.811	2.58809
1.998	0.2795	2.0036	0.18	2.1534	7.47654
3.3323	25.6498	4.4819	34.457	3.3429	25.4133
6.6695	0.16219	6.6587	0.1195	6.6624	0.05557
3.6626	0.16899	3.6688	0.05818	3.7458	2.09878
5.3262	6.53465	4.9995	0.01	5.1185	2.38024
5.6634	4.97173	5.9597	5.17118	5.786	2.91458
2.0004	0.005	2.0003	0.015	2.0184	0.90486
5.9873	0.29143	6.0048	0.08	5.6468	5.9619
5.9956	0.09165	6.0011	0.01833	6.2128	3.52769
2.6627	376.1	0.39644	136.165	2.7219	382.24
8.6151	30.514	6.6009	0.9865	6.7734	2.61328
5.9955	0.00834	5.996	0.06667	6.1901	3.23716
3.6674	0.17208	3.6611	0.15182	3.8118	4.11625
4.3275	0.15689	4.3343	0.02231	4.4713	3.16083
6.9982	0.01572	6.9971	0.04143	7.1148	1.68213
3.6703	26.6131	5.0013	0.026	5.0784	1.5416
4.9913	0.02203	4.9924	0.152	5.0686	1.52632
6.9927	0.0386	6.9954	0.06571	10.5844	51.3051
1.132	15.4024	1.3381	0.3575	1.3184	1.47224
7.1134	1.64759	6.9981	0.02714	7.1607	2.32349
3.9982	0.045	4	0	4.0227	0.5675
2.3694	135.34	1.0068	0.68	1.0143	0.74493
5.66	0.01943	5.6611	0.09824	5.7015	0.71364
4.9921	0.0822	4.988	0.24	5.1098	2.44186
5.0037	0.21631	4.9929	0.142	3.0254	39.406
3.6661	23.5251	2.9679	19.0573	3.6893	24.3067
5.3354	0.0375	5.3334	0.00125	5.1975	2.54809
2.3289	0.29967	2.3359	0.11	2.4383	4.38375
4.3259	0.21452	4.3352	0.04308	4.2599	1.73694
6.9966	0.15317	6.9859	0.20143	6.9274	0.8374
5.3067	32.6476	4.0006	0.015	4.0192	0.46493
5.9911	0.02336	5.9925	0.125	6.1819	3.16062
4.9941	0.18587	5.0034	0.068	4.1382	17.2922
4.989	0.09812	4.9939	0.122	5.0599	1.32161
7.7848	14.3932	6.8053	70.1325	4.1779	38.6081
6.661	4.689	6.9887	0.16143	7.0891	1.4366
0.1218	93.9127	2.0009	0.045	1.9982	0.13494
0.9985	0.78498	1.0064	0.64	1.0099	0.34777
5.095	6.87771	5.4713	173.565	5.4029	1.25016
5.9923	0.04004	5.9947	0.08833	6.1656	2.85085
4.6633	11.0944	4.1976	10.0514	4.78	13.8746
2.3337	0	2.3337	0.01571	2.3436	0.42422
4.5443	1.4914	4.6131	7.738	5.0943	10.4312
1.0001	19.0596	1.2356	23.56	1.4754	19.4076
3.9998	0	3.9998	0.005	4.0084	0.21501
5.3355	0.08801	5.3402	0.12875	5.5274	3.50549
3.9997	0.0025	3.9998	0.005	4.0068	0.17501
4.9971	0.06599	5.0004	0.008	5.1422	2.83577
3.9997	0.005	3.9995	0.0125	4.0038	0.10751
6.658	0.06604	6.6624	0.064	6.8334	2.56664
2.0004	0.03498	2.0011	0.055	1.9964	0.23487
2.0004	0.03498	2.0011	0.055	1.9964	0.23487
5.9889	0.09342	5.9945	0.09167	6.0777	1.38794
4.9963	0.02801	4.9977	0.046	5.1634	3.31553
2.8194	40.8784	2.0013	0.065	2.007	0.28481
3.328	27.671	4.6012	38.036	3.4625	24.7479
2.9907	0.26678	2.9987	0.04333	3.0731	2.48108
5.1544	6.47607	4.8409	3.182	5.0343	3.99512
4.1999	10.0105	4.6671	0.00929	4.7421	1.60699
5.9969	0.02501	5.9984	0.02667	5.9558	0.71019
4.5547	8.79473	4.9939	0.122	5.1845	3.81666
4.9926	0.14001	4.9996	0.008	5.1457	2.92223
4.306	13.8507	4.9983	0.034	5.1248	2.53086
5.523	7.94847	5.9999	0.00167	6.0643	1.07335
2.0008	0.04	2	0	1.9941	0.295
5.9856	0.18843	5.9969	0.05167	6.0698	1.21563
5.3476	6.94772	5.0002	0.004	5.0856	1.70793
**Average**	9.55988	**Average**	7.23948	**Average**	3.79577
**RMSE**	0.9121	**RMSE**	0.5165	**RMSE**	0.2714

**Fig. 4:**
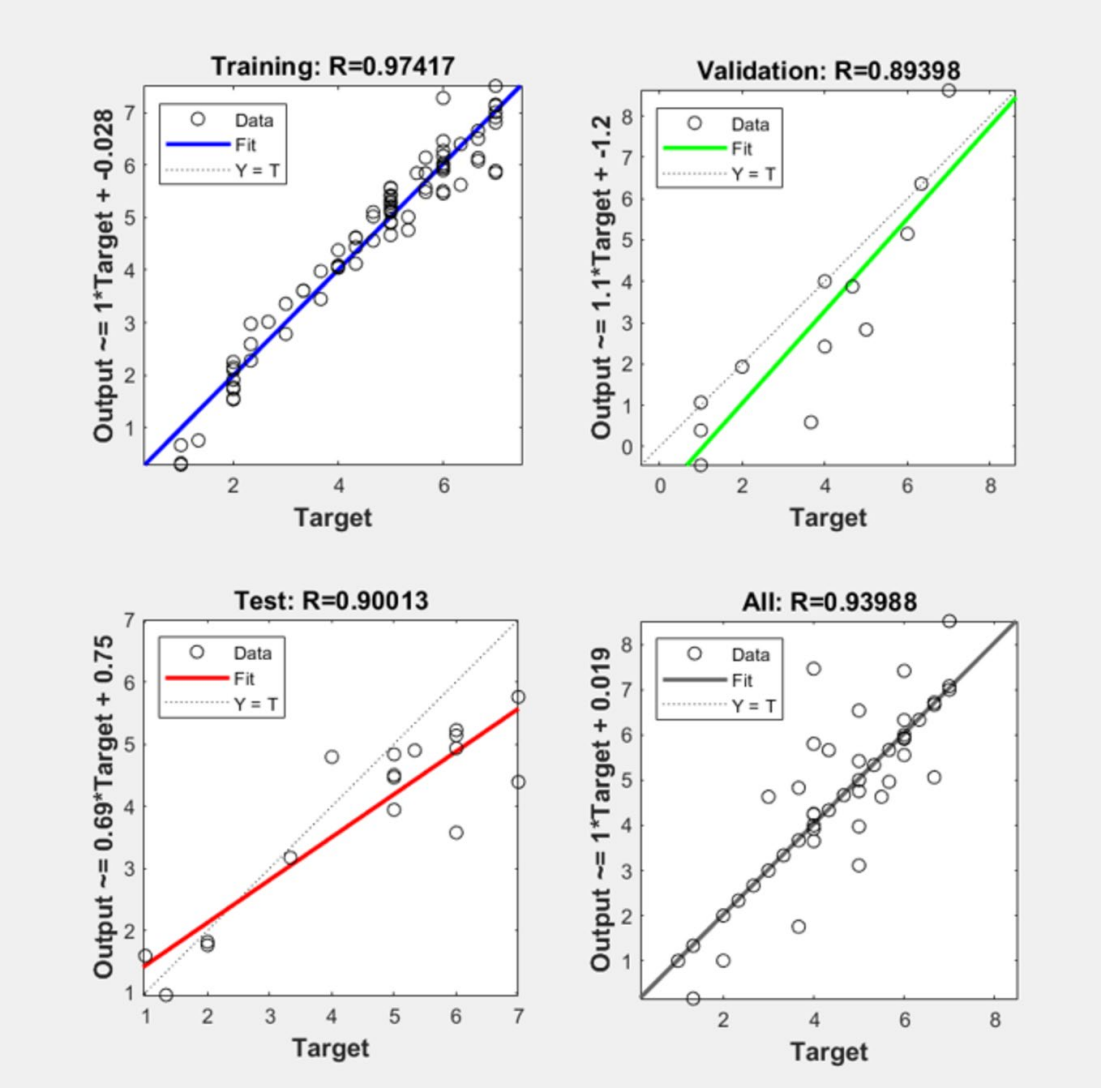
Validation Loss Rate.

Running the training and validation loss rate, acceptable results were seen with no under(over)fitting. Presented in Fig. 4 is the result of the test with 90% for the r-squared value of test output, 97% for training, and 89.40% for validation tests. An overall 93.99% rate was obtained.

### Validation

Following the study of Arpaci et al.^50^, the normalized score of importance (SHAP Package) was utilized in this study for the further verification of the sequential influence of a latent variable for actual use. Presented in Table 5 are the scores for this study. It could be seen that the results of the MLE are consistent with subjective norm, voluntariness, perceived ubiquity, and perceived usefulness as top latent variables influencing actual use of cloud storage in the Philippines.

**Table 5:** Normalized score of Importance.

Factor	Importance	Normalized Importance
SN	0.105	100%
VOL	0.104	99.4%
UB	0.103	98.5%
PU	0.097	92.8%
PB	0.091	86.9%
BI	0.089	84.8%
PEOU	0.084	80.6%
EX	0.083	79.2%
PC	0.083	79.2%
PR	0.083	79.0%
JR	0.080	76.1%

## Discussion

From the results, it could be deduced that subjective norms played the most significant factor affecting actual use of cloud storage in the Philippines. Based on the measured items, people around the individual performing a specific job influenced them in using the technology (i.e., classmates, workmates, family, and friends). It could be posited that both personal and professional usage of cloud storage has been considered by users. In addition, it was indicated that the user’s organization influenced them in using the cloud storage. As support, Yuduang et al.^28^expounded on the effects of other people around the user present a significant effect, which may go either positively or negatively. Campbell and Russo^52^explained that the compatibility of the work being done with the use of technology is important. To which, if people around them have experienced it and have positive feedback, users would most likely have high actual use or behavioral intentions to use the technology^53^. Thus, it could be deduced from the findings that users agreed with the use of cloud storage due to other people’s perception of its utilization.

Voluntariness also showed as one of the highest contributing factors (99.4%). Despite the presented influence from others, users were also seen to voluntarily use the cloud storage because their boss/teacher does not require them to use it; their school/organization, and work does not require them to use it as well. Due to people’s readiness to use technology nowadays, the adoption of which is relatively convenient for them^54^. When positive outcome is seen among users when using the technology, voluntariness would readily have a positive effect on actual use^55^. Contrary to the findings of Yuduang et al.^10^, they indicated that users did not present a positive relationship of technology use to actual use due to the forced implementation of system utility. In relation to this study, cloud storage is available online which has been capitalized by users for both personal and work-related use.

Perceived ubiquity, as defined is the overall availability of the system and could be utilized anywhere with any device, has been seen to have an effect on actual use. Users have indicated that their daily tasks are not interrupted by using cloud storage, can be accessed anytime and anywhere, and can help them achieve their responsibility compared to other technologies available. People’s recognition of the available technology would lead to their practice and develop the habit of using the system^56^. With this, it could be presented that recognition of the technology available led to their knowledge in how convenient, accessible, and flexible the cloud storage is. An adoption to the system features would lead to a more positive actual use of a system^54^.

Perceived usefulness was seen to be the fourth highest latent variable influencing actual use (92.8%), followed by perceived benefit (86.9%). Effectivity, efficiency, increase in productivity, file linkage and file sharing, and timeliness prompted the significant effect of perception in usefulness. When it comes to the benefits, data recovery, secured data services, and server maintenance presented a significant influence on actual use. In addition, compared to physical storage of data such as hard drives, cloud storage was seen to be more space saving. Contrasting to the study of Sohn and Kwon^57^, resistance on use of technology was seen, leading to a negative effect on actual use. However, the benefits of a technology at hand, such as that of cloud storage could be advantageous and people have seen it to be useful which would lead to actual use. As the performance promoted beneficial to the needed task^58^, and the system being highly useful^59^, actual use would be evidently positive due to enabling technologies^10,60^.

Behavioral intentions and perceived ease of use were also seen to be significant latent variables. Respondents indicated that they would consider cloud storage for work and personal storage, given more budget would also promote buying more storage, and it was seen that continuous intention on usage will be promoted. In addition, they have seen that cloud storage is easy to use and operate, achieve what they need to do, and highly convenient. These findings align with Golightly et al.^61^, explaining that people have adopted the use of cloud storage among various institutions, even in the workplace. The review of Hassan et al.^62^also provided justification on the measured items representing both latent variables. When people are satisfied with the system being used, the more likely a positive behavioral intention would be seen^28^. However, the habit developed in using a system would promote a lower significant score among perceived ease of use since users are mindlessly using the technology at hand due to adoption.

Aligning with the perceived ease of use, experience was seen to promote a significant effect on actual use. People have indicated that they are experienced in using the technology, interested, and have no difficulties with cloud storage. Technology self-efficacy may align with the findings under user experience in the use of cloud storage^28^. With the ability to accomplish any task given with the help of the technology, Meuter et al.^63^ indicated that development of experience would positively influence actual use. On the other hand, Talukder et al.^64^ expressed that technology fear may be present which would lead to a negative actual use and behavioral intention among users. Since cloud storage provides free storage, and that any additional storage would need more data which should be at a cost, user’s experience in using the system led for them to manage the existing storage for their convenience.

Both perceived cost and perceived risk had a significant effect on actual use. Low potential for data loss was indicated with considerable risk, trust in the system being utilized, and encountered little to no financial loss in using the technology. When cost is perceived to be reasonable, people are still encouraged to use the technology^63^. In accordance with perceived risks, people in the capital of the country in the Philippines – which is most of the respondents have been indicated to have high intentions in using technology for their benefits^20^. In the case of the results, people showed that they trust the system with the need for little to no information upon using the cloud storage. In addition, with low cost and free data storage available for different platforms, people less likely considered the two factors to be highly significant. Only when needed for more storage is cost affecting people’s perception^27^.

Lastly, job relevance was seen to be least among all factors because users feel that using it is important for their work/study, convenient in file sharing, data analysis, reporting and information, and storage. This is basically the intention of developers for cloud storage utility^24^. The more relevant the technology to a job, the more it has a positive influence on the user’s actual use^28^. However, in this study, it could be seen as the least significant since respondents showed that willingness to use due to convenience is the reason for cloud storage, rather than being aligned with the job relevance. In this case, it is more on their convenience for data storage and file transfer which is why relevance to the job is least likely significant in this study but is still considered significant. Therefore, with all latent variables being significant, it could be posited that people would continuously use cloud storage for both personal and work-related tasks, more usage compared to physical device storage, and would like to continue using the technology, similar to that of Tam et al.^60^ in measuring continuance intention to use mobile applications.

### Theoretical implications

From a theoretical standpoint, the integration of VF to the extended TAM showed a holistic measurement on actual use of technology. To which, both positive and negative perspectives were taken into account. The limited research insights on the behavioral intention and actual use of the continuous development of cloud computing and cloud storage have been established in this study, specifically focusing on developing countries like the Philippines. Moreover, it could be posited that complex insights could be obtained from the different variables included from the integrated framework. This could not be achieved with TAM due to limited structure, covering only the influence of technology adoption. In accordance, the perception of both gains and losses through the VF was obtained in relation to actual use of technology. On another note, extending the TAM provided better insights on other influential factors affecting actual use of cloud storage and services. Provided with the influence of others, benefits, convenience, and purpose of the technology that affected user’s work/study have been the reason why continuous intention to actually use the cloud storage was seen. Compared to other studies, people in the Philippines showed little significance on perceived risk in using cloud storage. This indicates that Filipinos worked around the available storage, its free usage, and its flexibility for their convenience. The findings of this study were able to limit the gap of cloud storage in relation to behavioral intentions and actual use. Since most technological development and assessment studies consider developed countries due to their marketability, developing countries should also be taken into consideration for their attempt to provide smart technologies and systems for advancement in development. Nonetheless, people in developing countries have been evidently using technology in everyday lives which should be considered by developers – showing that perceived usefulness and ease of use, relevance, and support should be considered.

Moreover, it could be argued that the use of machine learning brought better insight in terms of significant findings, influence, and significance of affecting variables and prediction. The neural network provided prediction of actual use while being able to categorize the effects of variables sequentially. The RFC helped in identifying consistent and highly influential factors. Both were the same and supported, which provided better insights and higher accuracy rates. Thus, it could be posited that the use of this methodology could help consumer behavior theory, consumer psychology, and technology acceptance assessment than the now considered traditional multivariate analyses. With the development of technology and adoption, machine learning algorithms were proven to be effective assessment tools for classification and variable identification. The growing popularity and utility of machine learning applications supports the claim that the assessment may be adopted and applied by future researchers.

### Practical implications and managerial insights

Basis on development and enhancement of cloud storage in developing countries may be implied with the results of this study. Since the Philippines aims to develop their cities to have more sustainable technological enhancement, the government may consider the findings of this study – evident on user’s adoption to available technology. The current cloud storage and cloud computing systems are an investment in the development of smart households, smart industries, and smart cities which could be capitalized by the government to help promote the current state of the country. With the promotion of technological advancement, automation and facilitation of infrastructure is needed which can be helped by the use of cloud computing and cloud storage. Results have shown that people are already aware of the technology, are accustomed to using it, and have the behavioral intention, better judgement and utilization of this may be considered by the government to enhance the current system utilized in the country.

Studies have found that both personal and work-related use of cloud storage is considered. Experience, voluntariness, and habit were developed with the technology and thus should be promoted by the government. Partnership with developers may be considered by the government. Among developers, local in-app advertisements may help reduce cost as well as gain profit with the same features and free storage. In this way, people would be able to recognize the benefit and use of cloud storage. At the same time, when almost all citizens utilize the technology, implementation for technology use by the government will not lead to anxiety and resistance among users. Lastly, further technological infrastructure is needed to be enhanced in the country for a more positive continuous intention.

### Limitations and future research

Despite available relevant findings and support, several limitations are still available for consideration. Future research may look at distinct representations of demographic characteristics. For example, which aspect of job/education is cloud storage being utilized. Second, it is also suggested for future researchers to consider knowledge and navigation of cloud storage or cloud computing to assess the maximization of use with technology. It is suggested that sophisticated clustering techniques may help in the analysis. Third, other factors may be considered and assessed since the limitations of this study are the extension of TAM and VF. A qualitative-quantitative mixed method may be conducted to encompass other factors not considered in this study. Separation of age group and its effect, location, technological capabilities, and knowledge may be applied by future researchers. In this way, more distinct practical and managerial implications may be created for developers to consider. Lastly, the conduction of SEM may be done for comparison and contrast of this study, justifying the outcome.

## Conclusion

Cloud storage has been widely considered and utilized among developed and developing countries due to its ability to provide a platform for large data and information storage. Its convenience, flexibility, accessibility, features, and functions have gained popularity for its development and enhancement. With different platforms gaining competitive advantages over others, the need to examine consumer behavior and user acceptance of actual use in developing countries should be explored to expand the knowledge among developers for promotion and technology advancement. This study analyzed the antecedents of actual use behavior of cloud storage in a developing country like the Philippines using a machine learning ensemble. With 616 valid responses, a total of 33,264 datasets were processed to analyze the actual use of cloud storage among Filipinos.

Results have presented consistent output of voluntariness, subjective norm, perceived benefit, perceived usefulness, and perceived ubiquity to be contributing factors affecting actual use behavior. Based on the measured items, people around the individual performing a specific job influenced them in using the technology (i.e., classmates, workmates, family, and friends). It could be posited that both personal and professional usage of cloud storage has been considered by users. In addition, due to people’s readiness to use technology nowadays, the adoption of which is relatively convenient for them. Experience, voluntariness, and habit were developed with the technology and thus should be promoted by the government.

From the findings, it could be deduced that when almost all citizens utilize the technology, implementation for technology use by the government will not lead to anxiety and resistance among users. Further technological infrastructure is needed to be enhanced in the country for a more positive continuous intention. In the theoretical implications, it was posited that the use of the integrated extended TAM and VF provided a holistic measurement of both negative and positive perspective of latent variables influencing technology acceptance, behavior, and actual use. Therefore, the application of the integrated framework may be used and expanded for other technology utilities in different countries.

## Electronic supplementary material

Below is the link to the electronic supplementary material.

Supplementary Material 1

## Data Availability

The datasets used and/or analyzed during the current study are available from the corresponding author on reasonable request.
