# Supplementary material for: Actual usage assessment among cloud storage consumers in the Philippines using a machine learning ensemble approach
Source: Sci Rep. 2024 Nov 22;14:28955. doi: 10.1038/s41598-024-80676-9 (PMC11584843; doi:10.1038/s41598-024-80676-9)
Supplement: Supplementary file 1 — Supplementary Material 1 [file 41598_2024_80676_MOESM1_ESM.docx]

**Questionnaire Items**

| Latent Variable | Item | Constructs | Reference |
| --- | --- | --- | --- |
| Subjective  Norm | SN1 | My boss/teacher influenced me to use cloud storage. | (Purnama & Ginardi, 2019) |
|  | SN2 | My colleagues/classmates influenced me to use cloud storage. | (Purnama & Ginardi, 2019) |
|  | SN3 | My family/relatives influenced me to use cloud storage. | (Yuduang et al., 2022) |
|  | SN4 | My friends influenced me to use cloud storage. | (Yuduang et al., 2022) |
|  | SN5 | My affiliation with an organization influenced my decision to utilize cloud storage. | (Yuduang et al., 2022) |
| Job  Relevance | JR1 | Use of cloud storage is important for my work/study. | (Purnama & Ginardi, 2019) |
|  | JR2 | In terms of information/file sharing, cloud storage helps my work/study. | (Purnama & Ginardi, 2019) |
|  | JR3 | In terms of data analysis, cloud storage helps my work/study. |  |
|  | JR4 | In terms of data reporting, cloud storage helps my work/study. |  |
|  | JR5 | In terms of storing information, cloud storage helps my work/study. |  |
| Perceived usefulness | PU1 | I think cloud storage helps me work/study effectively. | (Purnama & Ginardi, 2019) |
|  | PU2 | I think using cloud storage increases my productivity. | (Park & Kim, 2014) |
|  | PU3 | Cloud storage enables me to archive and retrieve my personal data faster. | (Burda & Teuteberg, 2014) |
|  | PU4 | Cloud storage enhances my effectiveness in archiving and retrieving my personal data. | (Burda & Teuteberg, 2014) |
|  | PU5 | Overall, I find cloud storage useful for my needed data. | (Burda & Teuteberg, 2014) |
|  | PU6 | I believe cloud storage simplifies my work/study (Example: Link now replaces huge file attachment in an emails). |  |
|  | PU7 | Time needed to upload and download data meets user’s requirement. |  |
| Perceived Ease  of Use | PEOU1 | Cloud storage is easy to use. | (Burda & Teuteberg, 2014) |
|  | PEOU2 | It is easy to get cloud storage to do what I want it to do. | (Burda & Teuteberg, 2014) |
|  | PEOU3 | Learning to operate cloud storage is easy | (Burda & Teuteberg, 2014) |
|  | PEOU4 | I believe it is easy and convenient to use since I can access cloud storage services anytime even via mobile devices. |  |
| Experience | EX1 | I am experienced in using cloud storage. | (Purnama & Ginardi, 2019) |
|  | EX2 | I have an interesting experience in using cloud storage. | (Purnama & Ginardi, 2019) |
|  | EX3 | I do not have any difficulties in using cloud storage. |  |
| Voluntariness | VOL1 | I voluntarily use cloud storage. | (Purnama & Ginardi, 2019) |
|  | VOL2 | My boss/teacher does not require the use of cloud storage. | (Purnama & Ginardi, 2019) |
|  | VOL3 | My work/school does not require to me use cloud storage. | (Purnama & Ginardi, 2019) |
|  | VOL4 | My affiliated organizations do not require to use cloud storage. |  |
| Perceived Ubiquity | UB1 | Use of cloud storage does not interrupt my other task (Example: Uploading/downloading files while running another program). | (Okazaki & Mendez, 2013) |
|  | UB2 | These services allow me to access data at the best moment for me. | (Okazaki & Mendez, 2013) |
|  | UB3 | Using these services outside my home or my workplace is not a problem for me. | (Okazaki & Mendez, 2013) |
|  | UB4 | When I use these services, I can achieve things that I cannot achieve in any other way. | (Okazaki & Mendez, 2013) |
| Perceived Benefits | PB1 | Recovery of data after disaster, power failure, or other unfortunate scenarios is not a hindrance. | (Yuduang et al., 2022) |
|  | PB2 | The data is saved in secured server. | (Yuduang et al., 2022) |
|  | PB3 | Cloud is space saving. | (Yuduang et al., 2022) |
|  | PB4 | I do not worry of server maintenance. | (Yuduang et al., 2022) |
| Perceived  Risk | PR1 | There is a low potential for loss involved in using cloud provider for work / personal archiving. | (Burda & Teuteberg, 2014) |
|  | PR2 | I accept any considerable risk involved in using cloud storage for work/personal archiving since it is not high. | (Burda & Teuteberg, 2014) |
|  | PR3 | A decision to use cloud storage for work/personal archiving is not risky. | (Burda & Teuteberg, 2014) |
|  | PR4 | I do not fear of cybercrime. |  |
|  | PR5 | I think it is not risky to give personal information when registering for cloud storage services. |  |
|  | PR6 | I think using cloud storage has low potential risks, even in payment transactions. |  |
| Perceived  Cost | PC1 | I may encounter little financial loss due to data loss. | (Wu et al., 2017) |
|  | PC2 | I may encounter little financial loss due to data leaking. | (Wu et al., 2017) |
|  | PC3 | I may encounter little financial loss due to unstable service. | (Wu et al., 2017) |
|  | PC4 | I am willing to pay significant amount monthly for cloud storage consumption. |  |
| Behavioral  Intention | BI1 | I would use cloud storage to archive my work and personal data. | (Burda & Teuteberg, 2014) |
|  | BI2 | I am very likely to archive my work and personal data using cloud storage. | (Burda & Teuteberg, 2014) |
|  | BI3 | I intend to use cloud storage for work and personal archiving in the future. | (Burda & Teuteberg, 2014) |
|  | BI4 | I will use cloud storage if I have budget. |  |
|  | BI5 | I will continue to use cloud storage if I have budget. |  |
| Actual  Use | AU1 | I always use cloud storage for my daily work / studies. | (Purnama & Ginardi, 2019) |
|  | AU2 | I use cloud storage more than physical storage devices (USB/HDD/SDcard/others). | (Purnama & Ginardi, 2019) |
|  | AU3 | I want to continue using cloud services |  |
